# The Use of Fetal Bovine Dermal Scaffold (PriMatrix) in the Management of Full-Thickness Hand Burns

**Published:** 2014-09-24

**Authors:** Alexis Lanteri Parcells, Jenika Karcich, Mark S. Granick, Michael A. Marano

**Affiliations:** ^a^Division of Plastic Surgery, Department of Surgery. Rutgers University New Jersey Medical School, Newark, NJ; ^b^The Burn Center at Saint Barnabas Medical Center, Barnabas Health, Livingston, NJ

**Keywords:** fetal bovine dermal scaffold, PriMatrix, hand burns, extracellular matrix, full-thickness burns

## Abstract

**Objective:** Management of full-thickness burn wounds represents a challenge when reconstructive options are not applicable. Fetal bovine dermal matrix is a bioactive collagen scaffold that assimilates into wounds and stimulates vascularization and dermal regeneration. **Methods:** We present the use of fetal bovine dermal scaffold PriMatrix in the treatment of a patient who sustained scald-immersion full-thickness burns of her bilateral hands that failed conventional wound therapy. **Results:** A 71-year-old woman with advanced Parkinson's disease sustained self-induced 5% mixed second- and third-degree scald-immersion burns of her bilateral hands and fingers. The patient underwent extensive debridement that resulted in partially avascular wounds measuring 66 cm^2^ and 72 cm^2^ with exposed extensor tendons and no evidence of bleeding. Meshed homograft was applied, but her hands remained partly avascular. PriMatrix fetal bovine dermal scaffold was applied to provide tissue remodeling over the bones, which allowed successful skin grafting and complete wound healing. **Conclusions:** Our experience shows fetal bovine dermal scaffold to be an effective method in management of complicated burn wounds in selected cases. Further studies need to be implemented to confer this conclusion.

The use of extracellular matrices (ECM) has increased in recent years due to their proven efficacy at treating a variety of complicated wounds. These scaffolds vary in source tissue and processing techniques. The host response similarly varies with this diversity,^1^^-^3 and this range of biological performance highlights the need for further understanding of in vivo characteristics of each product. Matching ECM with specific wound characteristics will aid in directing optimal utilization.

Fetal bovine dermal scaffold (FBDS) is a bioactive, regenerative collagen matrix that promotes wound healing through neovascularization.^1^^-^3 Clinical observations have demonstrated FBDS assimilation into a variety of wound types and promotion of vascularized tissue. The neodermis that develops at the implant site is capable of supporting a split-thickness skin graft, or it can undergo reepithelialization via keratinocyte proliferation.^4^ PriMatrix (TEI Biosciences Inc, Boston, Massachusetts) is a type of fetal bovine dermal substitute that has been approved by the Food and Drug Administration for treatment of diabetic and surgical wounds, as well as venous stasis ulcers and pressure sores. We present the use of PriMatrix in the treatment of complicated bilateral hand burns.

## MATERIALS AND METHODS

An ECM scaffold must promote host cell infiltration and remodeling to form functional tissues without an immunogenic response to be effective. The ideal matrix should be biocompatible, inexpensive, free of potential pathogens, and easy to apply.

When compared with postnatal wounds, fetal wounds have been found to display decreased inflammatory cells and increased production of collagen and fibroblasts.^5^ FBDS is composed primarily of type I and type III collagen preserved in its native, nondenatured state.^1^ Type III collagen, a form of fibrillar collagen in fetal dermis, is approximately 30% of its total collagen, whereas in adults skin it comprises 10% or less^6^^-^8 as the ratio of type I to type III collagen increases.^9^ This collagen has been reported to contribute to tissue regeneration and scarless wound healing,^10^^-^12 and wound healing experiments with adult mice genetically deficient in type III collagen have demonstrated increased scar formation and decreased tissue regeneration.^13^

Collagen is an optimal protein for cell proliferation due to its strong biochemical affinity for growth factors and angiogenic cytokines naturally present in human plasma. When FBDS comes in contact with the wound bed, the matrix binds host cells and growth factors, stimulating revascularization and dermal regeneration.^7^^,^^14^

FBDS is intrinsically strong and its mechanical properties approximate native dermis. Neill and colleagues^15^ analyzed a series of full-thickness wounds treated with FBDS that successfully reepithelialized with a split-thickness skin graft. Biopsies taken from the meshed dermal repair scaffold before grafting showed FBDS was revascularized and histologically similar to the dermis.

Although the time to reepithelialization is unknown, gross and histologic observations of FBDS in vivo demonstrated an inflammatory response peaked by day 7 and completely resolved by day 14. The FBDS transformed to a density and organization resembling native dermis by day 28. In addition, a progressive increase in intra-implant neovascularization was observed, with the greatest vessel count detected at day 28.^16^ The observed persistence of bovine-specific collagen fibers indicated the implant was remodeled but not entirely replaced with host collagen.

PriMatrix is highly porous and not artificially cross-linked, which likely contributes to rapid cell infiltration.^7^ All lipids, fats, carbohydrates, and noncollagenous proteins are removed during matrix processing. The product is terminally sterilized, stored at room temperature, and may be rehydrated in saline before implantation.

## RESULTS

A 71-year-old woman with a history of advanced Parkinson's disease sustained 5% mixed second- and third-degree scald-immersion burns to her bilateral hands. She was transferred to a regional burn center and underwent extensive debridement and eschar excision. The wounds on the dorsum of her hands were 66 cm^2^ and 72 cm^2^ with exposed extensor tendons and no evidence of bleeding.

**Figure 1:**
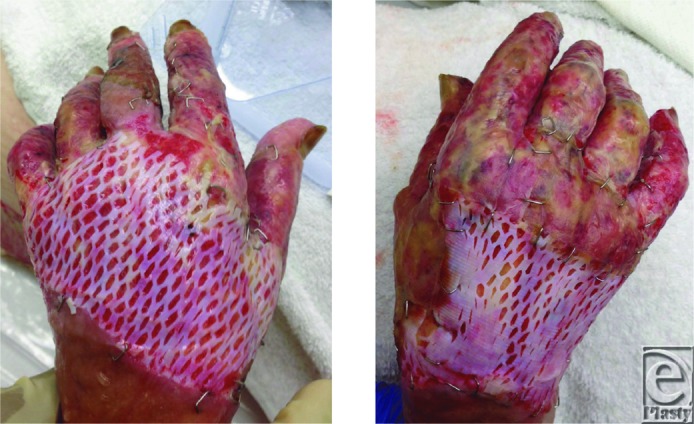
Bilateral hand wounds 5 days after application of fetal bovine dermal scaffold.

**Figure 2:**
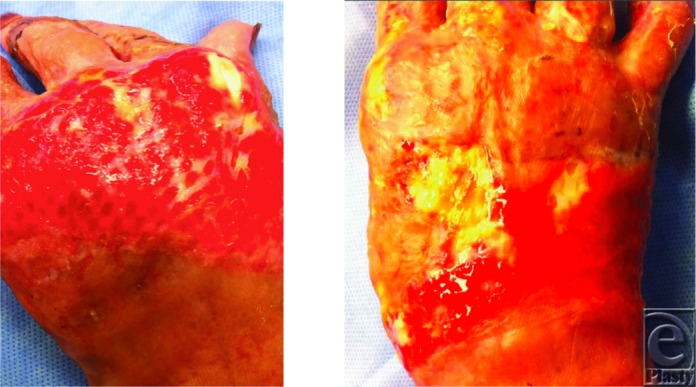
Bilateral hand wounds 21 days after application of fetal bovine dermal scaffold.

**Figure 3:**
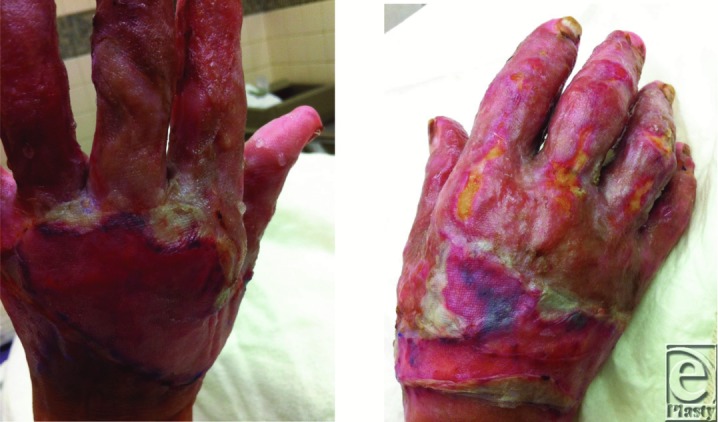
Bilateral hand wounds 5 days after application of split-thickness skin grafts.

Meshed cadaveric homograft was initially applied after escharotomy to protect underlying structures and promote granulation tissue for future autografting. After homograft excision, several areas of her hands remained partially avascular. PriMatrix dermal substitute was applied to provide protection and promote neovascularization for future skin grafting. The patient's wounds were unveiled 5 days after PriMatrix placement, and granulation tissue was present over the prior avascular extensor surfaces (Fig 1). Daily dressing changes ensured a moist wound environment (Fig 2). She underwent split-thickness skin grafting 2 weeks after PriMatrix application (Fig 3). Her wounds completely closed, and the patient was discharged to rehabilitation to continue physical therapy.

## DISCUSSION

Management of complicated wounds with large areas of exposed bone or tendon is challenging, especially if free flap or other complicated reconstructive surgery is not feasible. Several authors have clinically evaluated the effect of PriMatrix on chronic diabetic wounds, neuropathic ulcers, and complex surgical wounds. This is the first reported case of PriMatrix application to treat hand burns.

Karr^17^ conducted a retrospective comparison of PriMatrix and Apligraf (Organogenesis Inc, Canton, Massachusetts) and demonstrated accelerated healing in the PriMatrix group for the treatment of refractory diabetic foot and venous stasis ulcers despite larger initial wound size. While initially expensive, the authors argue that PriMatrix is cost-effective due to shorter healing times, decreased treatment visits, and fewer complications.^18^^,^^19^ Likewise, a study by Kavros^20^ showed PriMatrix to be efficacious in accelerating healing of 20 chronic neuropathic ulcerations compared to daily local wound care with Hydrogel and Xeroform gauze. There was no statistically significant difference in demographic and risk factors or severity of Charcot neuroarthropathy between the 2 groups. The benefit of healing neuropathic ulcerations quickly reduces the risk of osteomyelitis and limb amputation.

Higgs described 2 cases of PriMatrix used to treat traumatic wounds. The first case of a finger crush and degloving injury demonstrated assimilation of FBDS over exposed tendon and bone and eventual nonsurgical reepithelializion.^21^ The second case reported successfully combining PriMatrix with negative pressure wound therapy to treat necrotizing fasciitis.^22^ In addition, Lullove reported the use of PriMatrix on 10 nonhealing traumatic podiatric wounds. After 1 or 2 applications of meshed PriMatrix, these wounds healed on average in 75 days.^23^

Although there are currently no prior reports on the effectiveness of PriMatrix on hand burns, Kohanzadeh et al reported the use of PriMatrix in 5 patients with complex soft tissue hand injuries. While some patients went on to have split-thickness skin grafting, none required flap reconstruction. All patients underwent physical therapy and regained hand function.^24^ The authors found FBDS to be a valuable alternative to flap surgery in treating complex hand wounds in select cases.

Several studies have compared the effectiveness of FBDS on different types of wounds. Strauss and Brietstein conducted a retrospective review of 58 wounds treated with PriMatrix. The study included chronic diabetic wounds, venous stasis and pressure ulcers, and traumatic and surgical wounds. More than 80% of wounds underwent complete wound healing by 12 weeks after a single application of the FBDS. The scaffold was successfully incorporated into wounds with exposed tendon or bone, and the majority of cases achieved closure through reepithelialization by endogenous wound keratinocytes.^8^ Surgical wounds and pressure ulcers all healed in 75% of cases, while diabetic and venous ulcers healed 50% to 60% of the time. Likewise, Hayn^4^ found that more than 80% of complex traumatic and surgical wounds treated with FBDS underwent complete healing. The authors advocate the use of FBDS as a useful adjunct in the treatment of complex surgical wounds.

Many burn centers utilize cadaveric homograft for protection and promotion of granulation tissue in the immediate postburn period. In patients who fail to produce abundant granulation tissue, flap surgery is the criterion standard technique for wound coverage and restoration of hand function. While flap surgery was initially considered in our patient, she was deemed a poor candidate based on the extent of injuries and her current mental state. Serial debridement, cadaveric homograft placement, and eventual skin grafting were planned.

Our patient initially underwent escharotomy and placement of cadaveric homograft. On repeat exploration, several areas over her extensor surface appeared partially avascular. The decision was made to apply PriMatrix in an effort to provide scaffolding for skin graft take. This treatment was successful likely because areas of healthy granulation tissue vascularized the dermal scaffold, which then served as a bridge to smaller partially vascularized areas of tendon and bone. While the clinical response of FBDS has not yet been compared to an acellular cadaveric dermal counterpart, Wainwright reported the use of AlloDerm (Lifecell Inc, Bridgewater, New Jersey) and split-thickness skin grafts on 2 patients who sustained full-thickness burn wounds. Both patients went on to completely heal and the authors suggest that the supplemental dermal allograft can improve the healing of a meshed autograft.^25^ After 1 application, our patient's hands had enough granulation tissue to support a skin graft. Future research comparing the effects of would closure and hand function using these 2 dermal scaffolds would be valuable.

It is also important to note that time to wound healing varies greatly on degree of burn injury and complexity of structures involved. Two weeks after cadaveric homograft placement, our patient did not have sufficient granulation tissue over her extensor surface. At that time, the decision was made to apply PriMatrix for wound coverage. The patient underwent bedside dressing changes for 2 weeks until the wounds had sufficient granulation tissue. Although it is feasible to skin graft earlier, we decided to wait to ensure the graft was fully incorporated over her extensor surfaces. There is currently no recommended time to wait between FBDS and skin graft placement, and most suggestions are based on clinical assessment. Our patient achieved successful skin grafting and her total time to healing from the initial burn injury was approximately 5 weeks.

**Figure 4:**
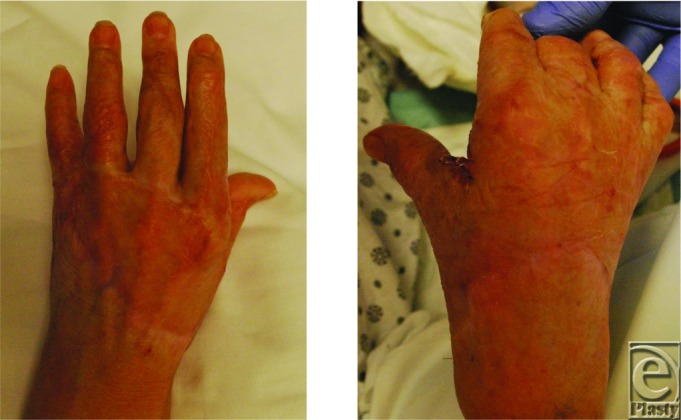
Bilateral hand wounds 9 months after application of split-thickness skin grafts.

While the initial primary goal in our burn patient was limb salvage and wound coverage, hand function is an important long-term goal. Our patient's hand burns healed completely with FBDS (Fig 4). However, despite splinting and aggressive physical therapy, diffuse scarring and significant contracture compromised her hand function. Other acellular scaffolds have been efficacious in treating burn contractures. A study by Askari and colleagues demonstrated healing of 9 patients with burn contractures treated with acellular dermal matrix. These patients experienced a significant increase in passive range of motion and web space lengthening.^26^ We attributed our patient's diffuse scarring to poor compliance due to her confused mental state. She eventually required surgical contracture release and local flap reconstruction 1 year after her initial incident. Her mental status has improved and she has gone on to achieve satisfactory functional recovery relative to the severity of the injury.

## CONCLUSIONS

Our case is the first to report the use of fetal bovine dermal substitute as a treatment for burn wounds in the hand. It is especially useful when conventional wound care methods fail or when flap surgery is contraindicated. Further investigation, preferably with a prospective clinical study, will be valuable in the future to identify the clinical benefits of FBDS. In addition, comparison studies on the effectiveness of various dermal matrices on burn wounds would be beneficial.
